# The Characterization of Microbiome and Interactions on Weathered Rocks in a Subsurface Karst Cave, Central China

**DOI:** 10.3389/fmicb.2022.909494

**Published:** 2022-06-29

**Authors:** Yiheng Wang, Xiaoyu Cheng, Hongmei Wang, Jianping Zhou, Xiaoyan Liu, Olli H. Tuovinen

**Affiliations:** ^1^State Key Laboratory of Biogeology and Environmental Geology, China University of Geosciences, Wuhan, China; ^2^School of Environmental Studies, China University of Geosciences, Wuhan, China; ^3^Department of Microbiology, The Ohio State University, Columbus, OH, United States

**Keywords:** subsurface biosphere, karst caves, phosphate-bearing minerals, microbial interaction, community assembly, co-occurrence network

## Abstract

Karst caves are a natural oligotrophic subsurface biosphere widely distributed in southern China. Despite the progress in bacterial and fungal diversity, the knowledge about interactions between bacteria, fungi, and minerals is still limited in caves. Hence, for the first time, we investigated the interaction between bacteria and fungi living on weathered rocks in the Heshang Cave *via* high-throughput sequencing of 16S rRNA and ITS1 genes, and co-occurrence analysis. The mineral compositions of weathered rocks were analyzed by X-ray diffraction. Bacterial communities were dominated by *Actinobacteria* (33.68%), followed by *Alphaproteobacteria* (8.78%), and *Planctomycetia* (8.73%). In contrast, fungal communities were dominated by *Sordariomycetes* (21.08%) and *Dothideomycetes* (14.06%). Mineral substrata, particularly phosphorus-bearing minerals, significantly impacted bacterial (hydroxyapatite) and fungal (fluorapatite) communities as indicated by the redundancy analysis. In comparison with fungi, the development of bacterial communities was more controlled by the environmental selection indicated by the overwhelming contribution of deterministic processes. Co-occurrence network analysis showed that all nodes were positively linked, indicating ubiquitous cooperation within bacterial groups and fungal groups, as well as between bacteria and fungi under oligotrophic conditions in the subsurface biosphere. In total, 19 bacterial ASVs and 34 fungal OTUs were identified as keystone taxa, suggesting the fundamental role of fungi in maintaining the microbial ecosystem on weathered rocks. *Ascomycota* was most dominant in keystone taxa, accounting for 26.42%, followed by *Actinobacteria* in bacteria (24.53%). Collectively, our results confirmed the highly diverse bacterial and fungal communities on weathered rocks, and their close cooperation to sustain the subsurface ecosystem. Phosphorus-bearing minerals were of significance in shaping epipetreous bacterial and fungal communities. These observations provide new knowledge about microbial interactions between bacteria, fungi, and minerals in the subterranean biosphere.

## Introduction

Karst caves are thought to be barren with few microorganisms and a weak reflection of the microbiology of surface soils due to oligotrophic conditions (Hess, 1900; Høeg, 1946; Caumartin, 1963; Palmer, 1991). Autotrophic microorganisms in caves usually obtain energy by chemosynthesis using inorganic energy sources (reduced Fe, Mn, and S compounds), organic and inorganic nutrients in host rocks, cave sediments, groundwater, and atmosphere (Jones and Macalady, 2016; Tomczyk-Żak and Zielenkiewicz, 2016; Hershey and Barton, 2018). The occurrence of various metabolic pathways is concurrent in cave microbiota (Kováč, 2018). In consideration of the oligotrophic conditions in caves, the microbial diversity encountered is surprisingly higher than expected as indicated by the application of molecular technology (routinely 10^6^ cells/g; Hershey and Barton, 2018).

Surfaces of various materials have been demonstrated to serve as important niches for microbial communities in diverse ecosystems, such as the surface of the painted murals, heritage buildings, petroglyph panels, and rock surfaces (Ma et al., 2015; Brewer and Fierer, 2018; Zhang et al., 2018; Irit et al., 2019; Ren et al., 2019; He et al., 2021). Rock surfaces are subjected to weathering by physical, chemical, and biological processes, thus offering special niches for microorganisms (Cañveras et al., 2001; Lian et al., 2008). Abundant radiation-resistant bacteria on the surface of limestone outcrops were detected, which were associated with lichens and grew under neutral to alkaline pH conditions (Brewer and Fierer, 2018). Usually, bacterial communities living on outcrop carbonate rocks exposed to sunlight and rainfall were dominated by *Actinobacteria*, *Proteobacteria*, *Chloroflexi*, *Bacteroidetes*, and *Acidobacteria*, whereas fungal communities consisted of *Eurotiomycetes*, *Lecanoromycetes*, *Dothideomycetes*, and *Leotiomycetes* (Choe et al., 2018). Microbial communities inhabiting the surfaces of carbonate rocks in permanently dark caves (Man et al., 2015; Yun et al., 2016; Zhu et al., 2021) are far less studied than those associated with outcrop rocks. Rock surfaces in subsurface caves are directly exposed to air, aerosol, and water vapor (high humidity), as well as a variety of physicochemical and biological dissolution–precipitation and oxidation–reduction reactions, leading with time to weathering and the formation of various secondary minerals (Aloisi et al., 2006; Gallagher et al., 2012). Rock walls act as surface biofilms for exchanges between the bedrock and the atmosphere. Microorganisms can use many minerals as sources of nutrients, carbon, and energy (Tomczyk-Żak et al., 2013). Nevertheless, the current knowledge is ambiguous on microbial communities in relation to mineral substrates and secondary minerals formed during rock weathering in subsurface caves.

Studies have shown that microbial communities in caves are affected by a variety of physical and chemical parameters. Studies in the Heshang Cave, a karst dolomite cave, have shown that pH and TOC are important drivers of the variations in bacterial communities among sediments, overlying soils, dripping water, and weathered rocks (Yun et al., 2016; Cheng et al., 2021). The underlying factors resulting in differences in bacterial and fungal communities living on rocks in caves are poorly understood. The physical properties and chemical compositions of minerals can profoundly impact the composition of microbial communities. Mineral phases in rocks serve as nutrients, substrates, and habitats for microbial communities (Shi L. et al., 2016). Some microbes use conductive mineral particles as conduits of interspecies electron transfer and cooperative catabolism (Kato et al., 2012). *Actinobacteria* are prevalent in Ca carbonate environments, and *alpha*-, *beta*-, and *gamma- Proteobacteria* are typically associated with complex minerals in cave environments (Barton et al., 2007).

Metabolic activities of microorganisms have important effects on the dissolution and formation of minerals. Moderately halophilic bacteria in liquid media cause the precipitation of calcite, Mg-calcite, and struvite (Arias et al., 2019). Carbonate biomineralization can be enhanced by ureolytic bacteria (Dhami et al., 2013; Arias et al., 2019). *Paracoccus versutus* XT0.6 isolated from the Xikuangshan antimony mine could dissolve stibnite and oxidize released Sb(III) to Sb(V), subsequently resulting in the formation of secondary Sb(V)-bearing minerals (Loni et al., 2020).

Highly diverse bacterial communities were detected from the weathered rocks in caves with the dominance of *Proteobacteria*, *Actinomycetes*, and *Firmicutes* (Yun et al., 2016; Zhu et al., 2019, 2021; Ma et al., 2021). In contrast, the diversity of fungi is relatively low due to the limited input of organic matter by photosynthesis in caves (Lee et al., 2012; Man et al., 2015). It was estimated that the content of organic matter in caves is three orders of magnitude less than that on the surface (Lavoie et al., 2010). Sunlight penetrates into the cave *via* the cave entrance to a limited distance and thus divides the cave into three zones: the photic zone with relatively strong light, the twilight zone with weak light, and the totally dark aphotic zone (Yun et al., 2016; Zhao et al., 2018). Due to the different light intensities, physical and chemical conditions vary in the three zones, resulting in different geochemical gradients and biological distribution patterns (Bonacci et al., 2009). In the photic zone, algae and green plants are visible on the rock surface and ground. Light is sufficient to support photosynthesis, thereby supporting CO_2_ fixation and primary production (Kozlova and Mazina, 2020). The availability of organic matter in the twilight and aphotic zones is extremely limited as it mainly depends on the input of exogenous organic matter *via* airflow, bat feces, and external organic matter excreted by burrowing animals such as cave rats (Pape and OConnor, 2014; Pfendler et al., 2019). Due to the differences in light, mineral components, and organic matter from different light zones, we hypothesize that (1) microbial communities living on the rocks vary with the zones, and mineral substrates significantly contribute to the variation; (2) bacterial and fungal communities interact intensively to sustain the microbial ecosystem on the rock; and (3) different ecological processes would contribute to the community assembly of bacteria and fungi, respectively.

To test these hypotheses, we collected weathered rock samples from different light zones in the Heshang Cave and subjected them to high-throughput sequencing of bacterial 16S rRNA and fungal ITS1 genes to investigate microbial communities. Variations in microbial communities and the driving forces responsible for these changes would be elucidated *via* the redundancy analysis. Interactions between bacterial and fungal communities were studied *via* co-occurrence network analysis to decipher how microbes interact with other microorganisms and sustain the ecosystem. Ecological processes and their contributions to the bacterial and fungal community assembly will be conducted to help understand how the specific microbial communities are established in caves. Our results will provide new insights into the interactions between bacteria and fungi living on carbonate rocks and the ecological processes responsible for microbial communities within subsurface caves.

## Materials and Methods

### Site Description and Sample Collection

The Heshang Cave, a dolomite karst cave, is 250 m long, 20–30 m wide, and 15–20 m high. It is located on the steep south bank of the Qingjiang River, northwest Hubei province, central China, with an elevation of 194 m above sea level (Supplementary Figure 1). An intermittent stream develops inside the cave during the rainy season, and dripping water is frequently observed from the ceiling of the cave all year round. The humidity is above 90% till saturated (Hu et al., 2008).

Weathered rock samples from the cave walls were collected along the cave from the entrance to the end on October 25, 2015. In total, nine sites were selected for sampling, with triplicates at each site (Supplementary Figure 1D), and the samples were designated as R1 to R9 accordingly. Green microbial mats were present on the surface of the cave walls close to the cave entrance (Supplementary Figure 2). At each sampling site, weathered rock samples were carefully scraped off with a sterilized scalpel and stored in 50 mL centrifuge tubes. The samples were placed on ice and transported to the Geomicrobiology Laboratory of China University of Geosciences (Wuhan) within 24 h and stored at –80 °C upon arrival until analysis. The wind speed and relative humidity were measured *in situ* with a handheld weather station (XYZ06E, Dalian Hede Technologies Corporation, China; Supplementary Figures 3A,B). The wind speed and relative humidity at each sampling site were continuously monitored for over 10 min. Values at 3, 6, and 9 min were read, and the mean values were used.

### Mineralogical Analysis

The weathered rock samples were freeze-dried (ALPHA 1-2 LD, Christ, Germany) and fully ground into powder. Mineral phases were analyzed by X-ray powder diffraction (XRD, Shimadzu XRF-1800) at a scanning speed of 0.05 s per step, with a step scan of 0.02° (10° ≤ 2 θ ≤ 90°) *via* a Cu target Kα radiation source (Ni filter, 40 kV, 40 mA). The phase composition and content of minerals in the samples were qualitatively and semi-quantitatively analyzed by Jade7 and X-powder software.

### DNA Extraction and Sequencing

Genomic DNA was extracted using a FastDNA™ SPIN Kit for soil DNA extraction (MP Biomedicals) in accordance with the instructions. The concentration and quality of extracted DNA were detected by using a micro-nucleic acid protein detector (Nanodrop 2000, Thermo Fisher). The primer set of 520F (5’-AYTGGGYDTAAAGNG-3’) and 802R (5’-TACNVGGGTATCTAATCC-3’) targeting the bacterial 16S rRNA V4 region (Claesson et al., 2009, 2010) and the primer set of ITS5F (5’-GGAAGTAAAAGTCGTAACAAGG-3’) and ITS1R (5’-GCTGCGTTCTTCATCGATGC-3’) targeting the fungal internal transcribed spacer (ITS1) region (Chang et al., 2001) were used for bacterial and fungal sequencing, respectively. All the sequences were conducted using an Illumina MiSeq PE250 platform (Shanghai Personal Biotechnology Co., Shanghai, China). All data are accessible *via* BioProject IDs of PRJNA337918 and PRJNA821722 for bacteria and fungi, respectively, on the NCBI.

### Data Processing and Statistical Analysis

FLASH (v1.2.7)^1^ was used for quality checking the original readings with Q30 and sequence assembly. Chimeras were removed by a DADA2 plugin in QIIME2 (2019.7, Quantitative Insight into Microbial Ecology). A table of bacterial amplicon sequence variants was constructed from the clean sequences with a 100% similarity, and the representative sequence of each ASV was obtained. Fungal OTUs were identified with a 97% similarity using Vsearch (2.15.1) software. All samples were resampled to the same sequencing depth. Bacterial annotation was carried out against the SILVA database,^2^ and fungal annotation was based on the UNITE database^3^ (Nilsson et al., 2019).

The microbial diversity index was calculated by a diversity plugin of QIIME2. The one-way analysis of variance (ANOVA) and Pearson correlation analysis were carried out using SPSS (v10.0). The heatmap analysis was performed using the OmicShare tools, a free online platform for data analysis.^4^ The vegan package of R software was used for NMDS analysis and output of rarefaction curves (Dixon, 2003), and the box diagram and stacked bar chart were visualized by the ggplot2 package of R software (Wickham et al., 2016). The Venn diagram was drawn using the plotrix package (Lemon et al., 2021). The redundancy analysis (RDA) of bacterial communities and physicochemical parameters was conducted by Canoco5 software. Indicator groups in the different light zones were obtained online with the interactive web pages^5^ by using the least discriminant analysis effect size (LEfSe) method, in which the linear discriminant analysis (LDA) threshold was set to 3.0. To reduce the network complexity, bacterial ASVs and fungal OTUs detected in at least three samples were selected. The relative abundance of retained bacterial ASVs accounted for > 36.6%, and the numbers of ASVs accounted for 6.6% of the total. The relative abundance of retained fungal OTUs accounted for > 94.4%, and the numbers of OTUs accounted for 15.2% of the total. The Gephi software (v.0.9.2) was used to visualize the co-occurrence network of bacteria and fungi. The ecological roles of each node were determined based on within-module connectivity (*Zi*) and among-module connectivity (*Pi*; Guimera and Amaral, 2005). Highly associative “hubs” in the microbiome can be identified from the network and are considered keystone taxa (Banerjee et al., 2018). Node topologies were classified as module hubs (highly connected nodes within modules, *Zi* ≥ 2.5 and *Pi* ≤ 0.62), network hubs (highly connected nodes within the entire network, *Zi* ≥ 2.5 and *Pi* > 0.62), connectors (nodes that connect modules, *Zi* < 2.5 and *Pi* ≥ 0.62), and peripherals (nodes connected in modules with few outside connections, *Zi* < 2.5 and *Pi* < 0.62; Deng et al., 2012; Shi S. et al., 2016). The assessment of ecological processes was performed with a mature workflow from the published literature (Stegen et al., 2012, 2013). The nearest taxon index (NTI), mean nearest taxon distance (MNTD), weighted β-mean nearest taxon distance (βMNTD), and weighted β-nearest taxon index (βNTI) were utilized to measure the phylogenic turnover using the theory of null model analyses across samples using the picante package (Kembel et al., 2010). Ecological processes were divided into deterministic processes with a |βNTI| > 2 and stochastic processes with a |βNTI| < 2. If βNTI values are above 2, variable selection is the crucial assembly process in the microbial community; otherwise, homogeneous selection dominates (βNTI < –2). To further specify the stochastic processes, the Raup–Crick matrix (RC_bray_) based on the Bray–Curtis matrix of microbial community was calculated using the vegan package. Dispersal limitation acting with the drift, undominated processes, and homogenizing dispersal were, respectively, dominant in bacterial communities under the conditions of RC_bray_ values > 0.95, | RC_bray_| < 0.95, and RC_bray_ < –0.95 (Stegen et al., 2012, 2013).

## Results

### Mineralogy, Wind Speed, and Relative Humidity in Heshang Cave

Minerals of the weathered rocks in the photic zone were dolomite, calcite, Mg-calcite, quartz, and amorphous materials (Supplementary Table 1). More minerals were detected in samples collected in the twilight zone, with the dominance of dolomite and quartz and a minor proportion of amorphous materials (Supplementary Table 1). Phosphate minerals (hydroxyapatite and fluorapatite), gypsum, and illite were also observed in twilight zone samples. Samples in the aphotic zone harbored eight minerals with Mg-calcite (18.6%) and phosphate minerals as the most abundant.

The average wind speed decreased from the cave entrance inward to the cave throughout the year. It was relatively stable (< 0.3 m/s) in the aphotic zone (Supplementary Figure 3A and Supplementary Table 2). The average relative humidity increased from inward to the cave and stabilized in the aphotic zone (>80%; Supplementary Figure 3B and Supplementary Table 3).

### Bacterial and Fungal Communities on the Weathered Rocks

A total of 6,885 bacterial ASVs and 5,320 fungal OTUs were obtained in this study, and 511 bacterial ASVs and 1,269 fungal OTUs were found in samples from photic, twilight, and aphotic zones as indicated by the Venn diagram (Supplementary Figure 4). The rarefaction curves of bacterial communities were leveled off across all sampling sites, whereas those of fungal communities at the R5 sampling site were rather steeper (Supplementary Figure 5).

**FIGURE 1:**
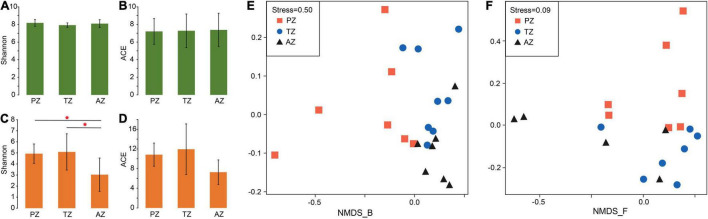
Alpha-diversity indices and non-metric multidimensional scaling (NMDS) analysis of bacterial **(A,B,E)** and fungal **(C,D,F)** communities of weathered rocks in the Heshang Cave, Hubei province, China. PZ: photic zone; TZ: twilight zone; AZ: aphotic zone. The mark with * indicates significant differences between the two groups (*P* < 0.05).

No significant differences were observed in the alpha diversity of bacterial communities among different zones. The Shannon indices varied between 7.56 and 8.12, and ACE values ranged between 4.18 and 10.60 (Figures 1A,B and Supplementary Table 4). The alpha diversity of fungi showed significant differences in the Shannon indices but not in ACE values between the three zones (Figures 1C,D and Supplementary Table 4).

The NMDS analysis showed poor interpretation of bacterial communities between the three light zones (stress > 0.2; Figure 1E), but clearly separated the fungal communities between the photic zone and the aphotic zone, while those in the twilight zone were scattered around (Figure 1F).

**FIGURE 2:**
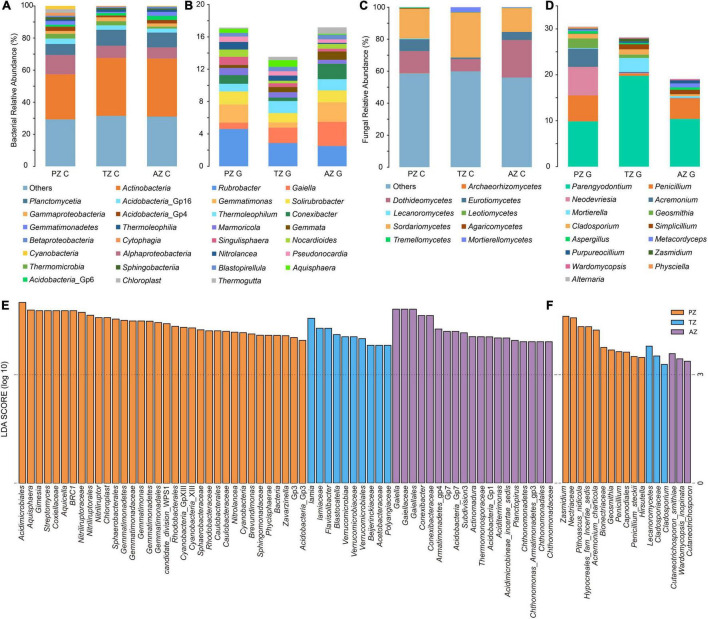
Bacterial **(A,B)** and fungal community **(C,D)** compositions of the top 15 classes **(A,C)** and genera **(B,D)** of weathered rocks in the Heshang Cave. Linear discriminant analysis (LDA) effect size taxonomic histogram with LDA = 4 of bacterial **(E)** and LDA = 3 of fungal **(F)** communities of the weathered rocks in the Heshang Cave, Hubei province, China. PZ: photic zone, TZ: twilight zone, AZ: aphotic zone. C and G after the sampling zone abbreviation indicate classes and genera of microbial taxonomy in the corresponding sampling location.

Taxonomically, 26 phyla and 55 classes of bacteria were detected with the dominance of *Actinobacteria* (relative abundance 33.68%). The most abundant class in all samples was *Actinobacteria*, followed by *Alphaproteobacteria* and *Planctomycetia*. The class of *Cyanobacteria* (4.31%) was only detected in the photic zone, which matched well with the presence of light (Figure 2A). At the genus level, *Rubrobacter* (4.61%), *Gemmatimonas* (2.25%), and *Solirubrobacter* (1.61%) ranked the top three in relative abundance in the photic zone. *Rubrobacter* (2.87%), *Gaiella* (1.92%), and *Thermoleophilum* (1.49%) were the three most abundant genera in the twilight zone, whereas *Gaiella* (3.00%), *Rubrobacter* (2.50%), and *Gemmatimonas* (2.41%) were the top three genera in the aphotic zone (Figure 2B). LEfSe identified *Cyanobacteria* (4.31%), *Gemmatimonas* (2.25%), *Nitrolancea* (0.94%), *Brevundimonas* (0.63%), and *Zavarzinella* (0.58%) as indicator groups in the photic zones with an LDA score of 3 (Figure 2E). Indicator groups in the twilight zone included *Aquisphaera* (0.83%), *Gimesia* (0.1%), and *Streptomyces* (0.58%), whereas *Conexibacter* (1.90%), *Actinomadura* (0.32%), *Aciditerrimonas* (0.35%), *Planctopirus* (0.16%), and *Chthonomonas* (0.19%) were indicator groups of the aphotic zone (Figure 2E).

Totally, 6 phyla and 21 classes of fungi were detected in all samples. *Sordariomycetes* was most abundant in photic (18.54%) and twilight zones (28.08%), followed by *Dothideomycetes* (13.74%) and *Eurotiomycetes* (7.43%) in the photic zone, *Dothideomycetes* (7.64%) and *Mortierellomycetes* (3.06%) in the twilight zone. The top three classes in the aphotic zone were *Dothideomycetes* (23.49%), *Sordariomycetes* (14.84%), and *Eurotiomycetes* (5.05%; Figure 2C). Among the top 15 genera, *Parengyodontium* ranked first in relative abundance among all samples. In the photic zone, *Neodevriesia* (6.23%) and *Penicillium* (5.64%) were the second and third abundant genera, whereas those in the twilight zone were *Mortierella* (3.06%), *Cladosporium* (1.15%), and in the aphotic zone, *Penicillium* (4.46%) and *Simplicillium* (0.97%) ranked second and third most abundant genera. To be noted, unidentified fungal genera in the aphotic zone accounted for a relatively high proportion (80.9%; Figure 2D). *Lecanoromycetes* (0.37%), *Hypocreales fam incertae sedis* (4.14%), *Bionectriaceae* (2.11%), and *Cladosporiaceae* (0.99%) were indicator groups in the photic zone, and those in the twilight zone were mainly *Nectriaceae* (0.15%) and *Zasmidium* (0.59%). The main indicator groups in the aphotic zone were *Cutaneotrichosporon* (0.05%) and *Wardomycopsis inopinata* (0.21%; Figure 2F).

### The Correlation Between Microbial Communities and Mineral Substrate

**FIGURE 3:**
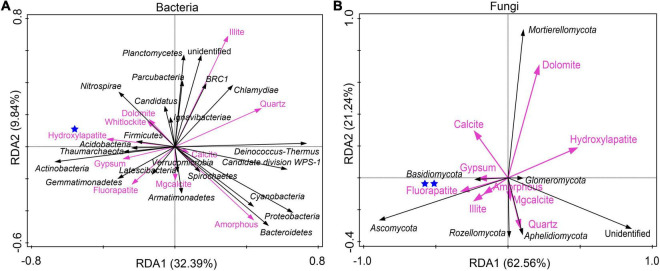
Redundancy analysis of mineral compositions and bacterial communities **(A)** and fungal communities **(B)** of the weathered rocks in the Heshang Cave, China. Mineral phases significantly impacting microbial communities were marked with one (with *P* < 0.05) or two blue stars (*P* < 0.01), respectively.

RDA was conducted to investigate the relationship between mineral phases and microbial communities living on the weathered rock. All minerals such as quartz, carbonate minerals (including dolomite, calcite, Mg-calcite, and aragonite), gypsum, illite, phosphate minerals (including white apatite, hydroxyapatite, and fluorapatite), and amorphous materials were included to elucidate their impact on bacterial and fungal communities. The results showed that hydroxyapatite and fluorapatite significantly affected bacterial and fungal communities with an explanation of 22.9% (*P* = 0.018) and 31.2% (*P* = 0.006), respectively (Figures 3A,B).

### Microbial Community Assembly on the Weathered Rocks

**FIGURE 4:**
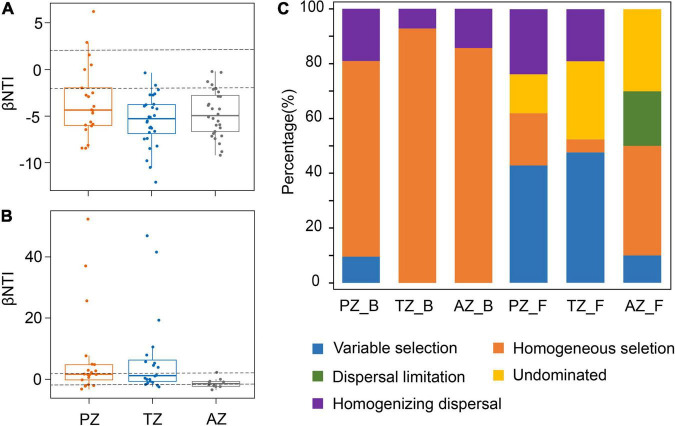
Phylogenetic and null model analyses show the assembly processes of bacterial **(A)** and fungal communities **(B)** in the photic zone (PZ), twilight zone (TZ), and aphotic zone (AZ) tested by βNTI values. The contribution of each ecological process to microbial community assembly in different zones in the Heshang Cave **(C)**. PZ_B, TZ_B, and AZ_B indicate the ecological processes responsible for bacterial community assembly in the photic zone, twilight zone, and aphotic zone, respectively, whereas those PZ_F, TZ_F, and AZ_F denote fungal community assembly.

The median of the βNTI matrix was below –2, indicating that the deterministic process controlled the assembly of bacterial communities (Figure 4A). Bacterial community assembly was overwhelmingly dominated by the homogeneous selection, with a contribution from 71.43 to 92.86% (Figure 4C). By contrast, the median of the βNTI matrix of fungal communities was between –2 and 2, indicating the dominance of the stochastic process in fungal community assembly (Figure 4B). Thus, ecological processes responsible for fungal community assembly were more complex than those for bacterial communities. Variable selection contributed 42.86 and 47.62% in the photic and twilight zones, respectively, but decreased to 10% in the aphotic zone, which was dominated by homogeneous selection (40%; Figure 4C). The contribution of dispersal limitation decreased inward to the cave from 23.81% in the photic zone, 19.05% in the twilight zone, to no contribution in the aphotic zone (Figure 4C).

### Interactions Between Bacterial and Fungal Communities Living on Weather Rocks

**FIGURE 5:**
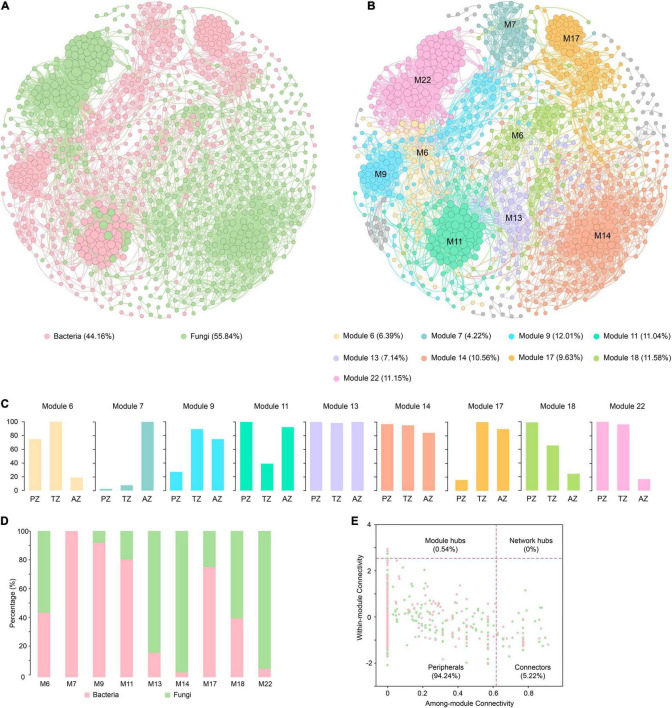
Co-occurrence networks of bacterial and fungal communities of weathered rocks in the Heshang Cave colored by bacteria and fungi **(A)** and by modules **(B)**. The proportion of ASVs/OTUs based on sampling zonation in each module **(C)**. The proportion of bacterial ASVs and fungal OTUs in each module **(D)**. The within-module connectivity (*Zi*)–among-module connectivity (*Pi*) plot shows distribution of ASVs/OTUs according to their module-based topological roles in the co-occurrence networks of bacterial and fungal communities **(E)**. Each dot represents an ASV in the dataset of bacteria (pink) or an OTU of fungi (green). PZ: photic zone, TZ: twilight zone, AZ: aphotic zone.

In total, 924 nodes and 7,425 links were observed in the co-occurrence network of bacteria and fungi with good modularity (0.788). Among the links, 800 connected fungal and bacterial nodes, 2,776 connected bacterial nodes, and 3,849 connected fungal nodes, respectively (Figure 5A). All nodes were positively linked. The average path length, diameter, and clustering coefficient were 5.634, 14, and 0.618, respectively.

Totally 28 modules were observed in the fungal–bacterial interaction network, and nine of the modules had a node relative abundance of over 4%, which were subject to further analysis (Figure 5B). Module 14 had the most nodes, accounting for 20.56% of the total nodes in the network. Nodes in modules 6, 18, and 22 mainly originated from PZ and TZ, whereas those in module 7 were mainly from AZ (Figure 5C). Nodes in modules 13 and 14 were evenly contributed by the three zones. Fungi dominated in modules 6, 13, 14, 18, and 22 (Figure 5D), and nodes in these modules were mainly affiliated with *Ascomycetes*. By contrast, bacteria dominated in modules 7, 9, 11, and 17 (Figure 5D), with the dominance of *Actinobacteria*. *Actinobacteria* and *Ascomycota* accounted for 19.59 and 16.99% of the total nodes, respectively.

Microbial keystone taxa were highly connected taxa that individually or in a guild exerted a considerable influence on the microbiome structure and functioning (Banerjee et al., 2018). Overall, 53 keystone taxa were identified (Figure 5E), five of them were module hubs (0.54% of all nodes), and the rest were connectors (5.19% of the total nodes). The module hubs and connectors accounted for 35.85, and 64.15% of all keystone taxa, respectively. Among 53 keystone taxa, 34 were affiliated with fungi and 19 were from bacteria. *Ascomycota* was the most dominant in keystone taxa, accounting for 26.42%, of which the most dominant class is *Sordariomycetes*, accounting for 15.09%, followed by *Actinobacteria* (24.53%). Module 13 harbored most keystone taxa, accounting for 22.64% of the total keystone taxa, followed by those in modules 6 (18.87%), 18 (13.21%), and 14 (13.21%).

## Discussion

### Variation of Microbial Communities on Weathered Rocks Along the Cave Passage

*Proteobacteria* and *Actinomycetes* were previously reported to dominate bacterial communities living on the weathered rocks (Tomczyk-Żak and Zielenkiewicz, 2016), which matched well with our observation of the dominance of *Actinomycetes* (37.66%) and *Proteobacteria* (12.68%) in the Heshang Cave. *Actinomycetes* can interact with a variety of mineral phases (Abdulla, 2009; Cuezva et al., 2012) and metals (Manimaran and Kannabiran, 2017) and are capable of degrading various organic compounds (Ningthoujam et al., 2012), which enables them to survive in extreme environments like caves. *Proteobacteria* (12.68%) ranked the second most abundant linage in the Heshang Cave, indicating highly diverse metabolic capabilities and important roles in the global carbon (Huügler et al., 2005; Kang et al., 2019; Schada von Borzyskowski et al., 2019), nitrogen (Shapleigh, 2011; De Mandal et al., 2017; Delmont et al., 2017), and sulfur cycles (Campbell et al., 2006; Marshall and Morris, 2013). The relative abundance of *Planctomycetes* (8.92%) in the Heshang Cave was relatively higher than that previously reported for other caves (<5%; Ortiz et al., 2013; Ma et al., 2015; Wu et al., 2015; Gulecal-Pektas, 2016), suggesting the uniqueness of bacterial communities in our study. The dominance of *Ascomycetes* (40%) in the Heshang Cave was consistent with previous reports on fungal communities in many other caves (Ogórek et al., 2013; Vaughan et al., 2015). Some of the fungal top 15 fungal genera observed in our study were also common in other caves, such as *Acremonium*, *Aspergillus*, *Cladosporium*, and *Penicillium* (Popović et al., 2015; Zhang et al., 2017; Zhang and Cai, 2019). *Parengyodontium* (13.66%) was the most abundant genus, which was frequently observed in other caves (Leplat et al., 2017, 2019). *Parengyodontium album* had often been the most abundant detected species when salt efflorescence was present (Ponizovskaya et al., 2019; Trovão et al., 2019). The high abundance of *Penicillium* (3.42%) may be related to bat activity (Karkun et al., 2012). The species *Penicillium janczewskii* is characterized by habitation in dry substrates depleted in organic matter, which is partially consistent with its presence in the cave (Mazina et al., 2020). *Neodevriesia* (2.37%) was reported as a dominant group on mural paintings in North Thai temples (Suphaphimol et al., 2022).

Alpha diversities and bacterial community compositions on the rocks did not show significant differences as indicated by Shannon and ACE indices and NMDS analysis in the sampling zones (the photic zone, twilight zone, and aphotic zone), suggesting a fundamental role of the rock substrate (carbonate especially), rather than sunlight in the shaping of bacterial communities. In contrast, the alpha diversity of fungal communities in the aphotic zone was significantly lower than that in the other two zones, which may be related to the isolation of the aphotic zone from light and plants. Fungal communities are known to be tightly associated with plant communities (Tedersoo et al., 2014; Purahong et al., 2018; Adamczyk et al., 2019), and almost 20% of total fungal species are symbiotic with lichens (Kirk et al., 2001; Honegger, 2009). Our results also found high fungal diversity at the entrance of the Heshang Cave, where lichens were observed on the rock surface. The decrease in fungal diversity inward to the cave may result from the impediment in the transfer of fungal spores due to the slow wind speed inside the cave (Supplementary Figure 3A). In fact, our observation of the significant positive correlations between the relative abundances of fungal classes such as *Wallemiomycetes*, *Agaricomycetes*, *Tremellomycetes*, *Lecanoromycetes*, *Exobasidiomycetes*, and the wind speed (Supplementary Figure 3C) also supported the decrease in the fungal diversity inward to the cave.

Despite small differences in bacterial communities, indicator groups were distinct in three light zones. The indicator groups in the photic zone were *Cyanobacteria*, *Gemmatimonas*, *Nitrolancea*, *Brevundimonas*, and *Zavarzinella*, which are closely related to light-dependent metabolism. Many cyanobacterial species can survive on soil and other terrestrial habitats and play important roles in the carbon and nitrogen cycles (Wegener et al., 2010; Forchhammer and Selim, 2020). Some species in *Gemmatimonas* such as *Gemmatimonas aurantiaca* can accumulate polyphosphate (Zhang et al., 2003), and other species like *Gemmatimonas phototrophica* are able to use light as an energy source (Zeng et al., 2015). *Nitrolancea* spp. are obligately aerobic, which are chemolithoautotrophic nitrite-oxidizers (Sorokin et al., 2014). *Flavisolibacter* are aerobic chemoheterotrophs in the phylum *Bacteroidetes* (Lee J. J. et al., 2016). The main indicator groups in the twilight zone were *Aquisphaera*, *Gimesia*, and *Streptomyces*. *Aquisphaera* and *Gimesia* belong to the phylum *Planctomycetes*. *Aquisphaera* forms large visible cell aggregates during growth, which may affect the survival of other microorganisms (Bondoso et al., 2011). Isolates of *Gimesia chilikensis* are salt- and alkali-tolerant (Kumar et al., 2020). Many *Streptomyces* species produce multiple antibiotics or other secondary metabolites, which act synergistically or contingently against biological competition (Challis and Hopwood, 2003). *Conexibacter*, *Actinomadura*, *Aciditerrimonas*, *Planctopirus*, and *Chthonomonas* were the indicator groups in the aphotic zone. They can survive in extreme environments, which is consistent with the lack of organic matter in the depths of the cave. *Conexibacter*, *Actinomadura*, and *Aciditerrimonas* all belong to class *Actinobacteria* and have been frequently isolated from extreme environments. For example, *Conexibacter stalactiti* was isolated from samples of stalactites collected from a lava cave (Lee, 2017), and *Actinomadura namibiensis* was isolated from desert sandy soil in Namibia (Wink et al., 2003). *Chthonomonas calidirosea* was thermophilic and isolated from geothermal soil (Lee K. C. et al., 2016; Compton et al., 2020). Microbes in the aphotic zone can also be involved in iron reduction. For example, *Aciditerrimonas ferrireducens* is capable of reducing ferric iron aerobically or anaerobically (Itoh et al., 2011; Hu et al., 2014).

The indicator groups of fungi also varied in the zones. In the photic zone, *Lecanoromycetes*, *Hypocreales*, *Bionectriaceae*, and *Cladosporiaceae* were identified as indicator groups. *Lecanoromycetes* is the largest class of lichenized fungi and includes most of the lichen-forming fungal species (> 13,500 species; Miadlikowska et al., 2006; Hofstetter et al., 2007). The majority of lichenized fungi in *Lecanoromycetes* are obligate mutualists, which can obtain organic nutrients from photosynthetic symbiotes and N compounds fixed by cyanobacteria (Gueidan et al., 2015; Jüriado et al., 2017). The *Bionectriaceae* is a family in the order *Hypocreales*, which tends to grow on plant materials (Khodosovtsev et al., 2012), and some species are related to algae (Khodosovtsev et al., 2012), bryophytes (Döbbeler et al., 2015), or other fungi. *Cladosporiaceae* is also associated with plants (Alghuthaymi, 2017) and is dominant in highly saline environments (Yang and Sun, 2020). The indicator groups in the twilight zone were *Nectriaceae* and *Zasmidium*. Numerous members in the family *Nectriaceae* (*Hypocreales*) are plant and human pathogens, and several species belonging to this family are used extensively in industrial and commercial applications for biodegradation and as biocontrol agents (Lombard et al., 2014, 2015). *Zasmidium cellare* has been reported in the Mammoth Cave, Kentucky (Barr, 1968), and is a strain known for living in dark and ethanol-rich environments (Cailhol et al., 2020). The indicator groups in the aphotic zone included *Cutaneotrichosporon* and *Wardomycopsis inopinata. Cutaneotrichosporon oleaginosus* is an oleaginous yeast with fast growth and also grows as filamentous fungus in the soil and leaf litter (Bracharz et al., 2017). Information on the function of *Wardomycopsis inopinata* in the environment is not available.

### The Impact of Mineral Substrate on Microbial Communities and Ecological Processes in Community Assembly

RDA indicated that bacterial communities were significantly affected by hydroxyapatite, mainly due to phosphate-solubilizing bacteria *Streptomyces* and *Pseudomonas.* An isolate of *Pseudomonas fluorescens* from the Heshang Cave has been demonstrated to solubilize fluorapatite, making phosphate bioavailable for microorganisms (Zhou et al., 2017). Fungal communities were positively impacted by fluorapatite, attributed to phosphorus-solubilizing soilborne fungi *Aspergillus niger* and *Penicillium* spp. (Reyes et al., 2007; Farhat et al., 2009; Li et al., 2016). The mechanism of phosphorus-solubilizing microorganisms is generally attributed to organic acid metabolites produced by bacteria and fungi (Kavanagh, 2005; Jones and Oburger, 2011). Phosphate is a common minor element in many silicate, sulfide, and carbonate minerals. Under phosphate-limited conditions in soil environments, microorganisms colonizing minerals may acquire bioavailable phosphate as it solubilizes from the mineral phase (Rogers and Bennett, 2004). Our observation of the significant impact of phosphate-bearing minerals on bacterial and fungal communities on the weathered rocks substantiates the development of microbial communities nutritionally dependent on the mineral-phase substrate.

Ecological processes responsible for the development of microbial communities were also investigated in the Heshang Cave. The assembly of bacterial communities is mainly a deterministic process, with the dominance of homogeneous selection across different sampling zones, indicating a spatial homogeneity in environmental conditions and the convergence in bacterial communities (Dini-Andreote et al., 2015). This conclusion is also consistent with the result of the NMDS (Figure 1E). In contrast, different ecological processes contributed to fungal assembly in sampling zones. Variable selection dominated the assembly of fungal communities in both the photic and twilight zones, indicating the strong environmental selection of fungal communities partially due to sunlight. Many fungal groups were associated with algae, cyanobacteria, and plant debris, all of which were highly dependent on sunlight. Homogeneous selection only dominated the fungal assembly in the aphotic zone, indicating that a homogeneous environmental condition resulted in the convergence in fungal communities. The occurrence of dispersal limitation in the aphotic zone indicates a weakening selection, which may have resulted from slow wind speed and high relative humidity toward the end of the cave entrance (Supplementary Tables 3,4). Slow airflow is known to decrease airborne transmission of fungal spores (Quintero et al., 2010), and fungal survival increases with relative humidity (Oliveira et al., 2005; Hameed et al., 2012). The relative abundances of fungal classes *Wallemiomycetes*, *Agaricomycetes*, *Tremellomycetes*, *Lecanoromycetes*, and *Exobasidiomycetes* showed significant positive correlations with the wind speed (Supplementary Figure 3C), and similar phenomena in air have also been reported (Begerow and Kemler, 2018; Els et al., 2019).

### Interaction Between Bacteria and Fungi Living on Weathered Rocks

The co-occurrence network of bacteria and fungi showed good modularity and positive links between nodes, indicating that the bacterial and fungal communities formed a close organization through cooperation among different species. Their interactions increased the complexity of the community structure and the stability of the ecosystem (Chen et al., 2018).

In total, 53 keystone taxa were identified, which usually interact with many other groups, thus promoting the exchange and flow of energy, information, metabolites, and nutrients among different species and maintaining the balance of microbial communities (Berry and Widder, 2014; Ma et al., 2016). There are many unknown functions of the keystone taxa in this study, but some clues can be garnered from the previous investigations. The fungal keystone taxa mainly belonged to classes of *Sordariomycetes*, *Dothideomycetes*, and *Eurotiomycetes*. Members of *Sordariomycetes* are ubiquitous in natural environments (Zhang and Wang, 2015). They participate in the decomposition of organic matter and nutrient cycling as saprotrophs in almost all ecosystems, and they include endophytes and pathogens of plants (Arnold and Lutzoni, 2007), arthropods (Samuels and Blackwell, 2001), and mammals (O’Donnell et al., 2010), and even as mycoparasites attacking other fungi (Broberg et al., 2021). *Sordariomycetes* also contain species known to produce secondary metabolites such as potent mycotoxins (Zhang and Wang, 2015). *Dothideomycetes* is the largest class of *Ascomycetes*, which includes numerous rock-inhabiting fungi, adapted well to nutrient-poor and dry habitats on rock surfaces (Ruibal et al., 2009; Haridas et al., 2020). *Eurotiomycetes* can degrade a wide variety of organic substrates and can tolerate high salinity, complete darkness, and oligotrophic conditions (Greiner et al., 2014; Zhang and Wang, 2015), which favor their survival in caves.

*Nocardioidaceae*, *Planctomycetaceae*, and *Rubrobacteraceae* were keystone taxa of bacterial classes in the network. *Nocardioidaceae* uses a wide range of carbon and nitrogen sources, including unusual organic compounds and toxic environmental pollutants, and has versatile metabolic pathways (Evtushenko et al., 2015). Like many other actinomycetes, *Nocardioidaceae* can survive under extreme conditions such as desiccation, low and high temperatures, UV damage, and toxic compounds (Kirby et al., 2006; Brodie et al., 2007; Griffin, 2007; Polymenakou et al., 2008). Members of the *Nocardioidaceae* are symbiotic with plants and may exist as reciprocal endophytes. They can also occur in association with lichens (Li et al., 2007) and fungi (Lauer et al., 2007). *Marmoricola* is a genus of *Nocardioidaceae*, which are found in different environments, such as marble, beach sediments, and agricultural soil (Lee, 2007; Freed et al., 2019). Some species are alkali-tolerant or display alkaliphilic properties (Evtushenko, 2015). The family *Planctomycetaceae* is distributed in both soil and water (Wiegand et al., 2018) and shows strong tolerance to seawater, acid peat bog, hot springs, and low temperature (Kulichevskaya et al., 2007, 2008; Fuerst and Sagulenko, 2011). It can use a special mechanism to absorb and digest complex polysaccharides, which may help it compete for nutrients in natural habitats (Boedeker et al., 2017; Schubert et al., 2020). The family *Rubrobacteraceae* is usually represented by its thermophilic and radiation-resistant types (Albuquerque et al., 2014). The genus *Rubrobacter* of *Rubrobacteraceae* is involved in chlorophyll biosynthesis (Gupta and Khadka, 2016). These keystone bacteria and fungi impact microbial community composition and symbiotic or parasitic relationships on rock walls. Some of their secondary metabolites have antibiotic or toxin properties that can promote or inhibit effector species and modulate interactions in microbial communities.

The co-occurrence network analysis provides us with a new way to find potential symbiotic or parasitic associations between bacteria and fungi. Bacteria can be endosymbiotic with fungi (termed endohyphal bacteria), which are phylogenetically and ecologically widespread. Endohyphal bacteria recently have been documented in diverse *Ascomycota*, including members of multiple classes (*Pezizomycetes*, *Eurotiomycetes*, *Dothideomycetes*, and *Sordariomycetes*; Hoffman and Arnold, 2010; Shaffer et al., 2016, 2017). Our network analysis found that many unannotated groups within *Eurotiomycetes*, *Dothideomycetes*, and *Sordariomycetes* serve as keystone taxa in the network, which are also tightly connected with bacteria. For example, *Betaproteobacteria* has a connection with unidentified fungi, and related studies have demonstrated that some genera of *Betaproteobacteria* can parasitize as endosymbionts in fungal hyphae (Hoffman and Arnold, 2010). *Xanthomonadaceae* has been found to subsist in fungal hyphae (Hoffman and Arnold, 2010), as also notices for unidentified fungi in our network. The observation of the connection between *Caldiline* and unidentified fungi in our study is consistent with a previous report of a negative correlation between *Caldiline* and an unclassified fungus of *Pezizomycotina* (Fouts et al., 2012). *Gammaproteobacteria* in the network are linked to many OTUs in *Dothideomycetes*, *Sordariomycetes*, and unidentified fungi. Arendt et al. (2016) demonstrated that some species of *Gammaproteobacteria* can be successfully introduced into *Dothideomycetes* and *Sordariomycetes*, thus substantiating their symbiotic relationships.

It should be emphasized that co-occurrence networks visualize the correlations between microbial groups, including real ecological interactions (such as reciprocity), and also include non-random processes (such as niche overlap). They do not, therefore, necessarily reflect direct interactions between the groups. Future experiments will assess whether the identified key species directly affect other members of the microbiome or indirectly affect the performance and fitness of the host, thus affecting other community members.

## Conclusion

We studied the ecological processes responsible for the community assembly, endeavoring to provide new insights into microbial interactions and establishment of microbial communities under extreme conditions in the subsurface cave biosphere.

1. Microbial indicator groups varied with different light zones, indicating functional changes in response to the light variation in karst caves. Phosphate minerals such as hydroxyapatite and fluorapatite significantly influenced bacterial and fungal communities living on weathered rock surfaces.
2. The co-occurrence network analysis showed that bacteria and fungi on rock surfaces were mainly positively linked, indicating extensive, close cooperations among microbial groups across different domains connected under stark environmental conditions to maintain the microbial ecosystem. The co-occurrence network may also provide us with a new robust way to decipher symbiosis and parasitism between bacterial and fungal groups.
3. Homogeneous selection dominated bacterial community assembly throughout the cave, resulting in the convergence of bacterial communities. Variable selection contributed most to the fungal community assembly in the photic and twilight zones, whereas homogeneous selection with dispersal limitation contributed more to fungal assembly in the dark zone.

## Data Availability Statement

The datasets presented in this study can be found in online repositories. The names of the repository/repositories and accession number(s) can be found below: https://www.ncbi.nlm.nih.gov/bioproject/337918, http://www.ncbi.nlm.nih.gov/bioproject/821722.

## Author Contributions

YW, JZ, and XL completed the sample collection. JZ finished DNA extraction. YW and XC completed data analysis, visualization, and original draft preparation. HW designed this experiment, supervised data analysis, acquired funding, and wrote and edited this manuscript. OT participated in data discussion and manuscript drafting. All authors contributed to the article and approved the submitted version.

## Conflict of Interest

The authors declare that the research was conducted in the absence of any commercial or financial relationships that could be construed as a potential conflict of interest.

## Publisher’s Note

All claims expressed in this article are solely those of the authors and do not necessarily represent those of their affiliated organizations, or those of the publisher, the editors and the reviewers. Any product that may be evaluated in this article, or claim that may be made by its manufacturer, is not guaranteed or endorsed by the publisher.

## Footnotes

1
http://ccb.jhu.edu/software/FLASH/

2
http://greengenes.secondgenome.com/

3
https://unite.ut.ee/

4
https://www.omicshare.com/tools

5
http://huttenhower.sph.harvard.edu/galaxy/
