# Supplementary material for: The Characterization of Microbiome and Interactions on Weathered Rocks in a Subsurface Karst Cave, Central China
Source: Front Microbiol. 2022 Jun 29;13:909494. doi: 10.3389/fmicb.2022.909494 (PMC9277220; doi:10.3389/fmicb.2022.909494)
Supplement: Supplementary file 1 [file Data_Sheet_1.docx]

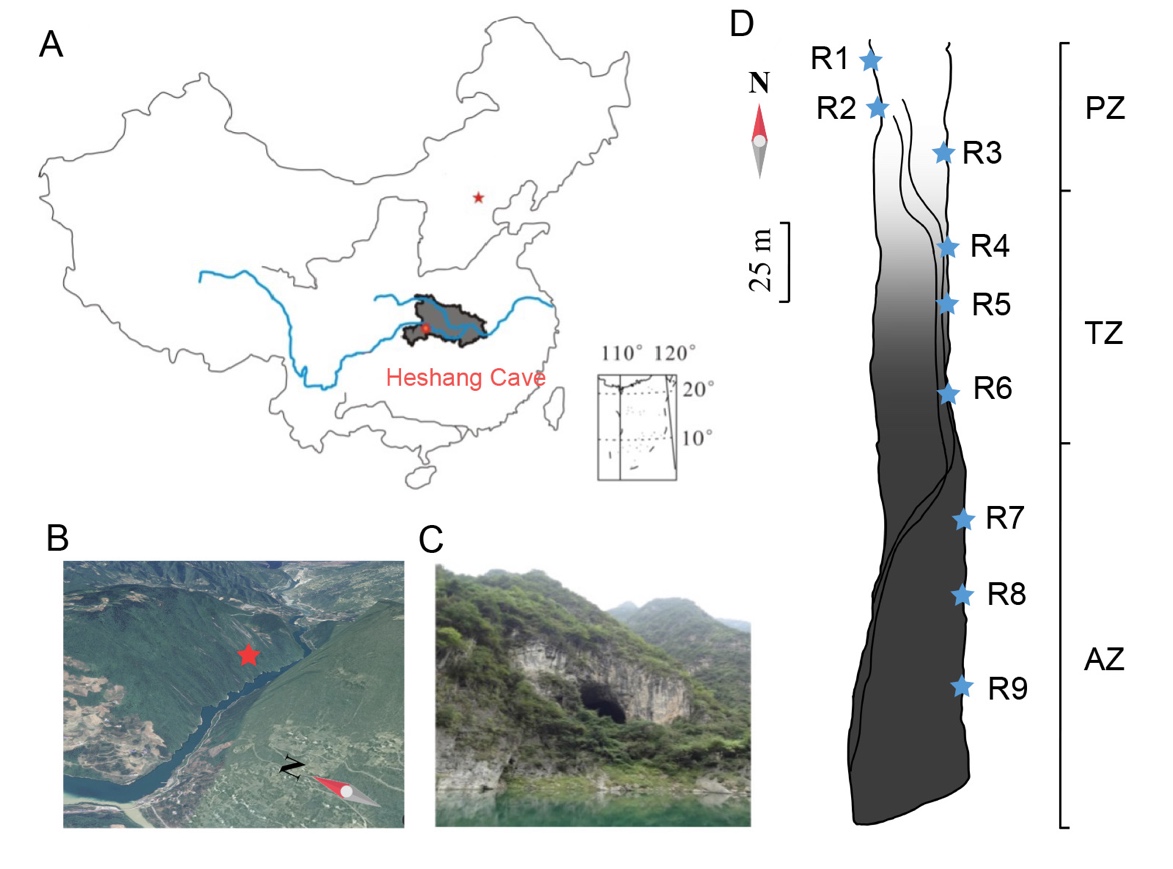


Supplementary Figure 1. Geographic location of the Heshang Cave (A). Satellite image of the Heshang Cave (from Google Earth) (B). Exterior view of the entrance of the Heshang Cave (C). Sampling sites inside the Heshang Cave (D). The photic zone (PZ) is close to the entrance with enough light to support photosynthesis. Twilight zone (TZ) locates in the middle of the cave, where sketch of objects can be seen hardly face to the entrance. Aphotic zone (AZ) is totally dark with bats living on the ceiling in the Heshang Cave, P R China.


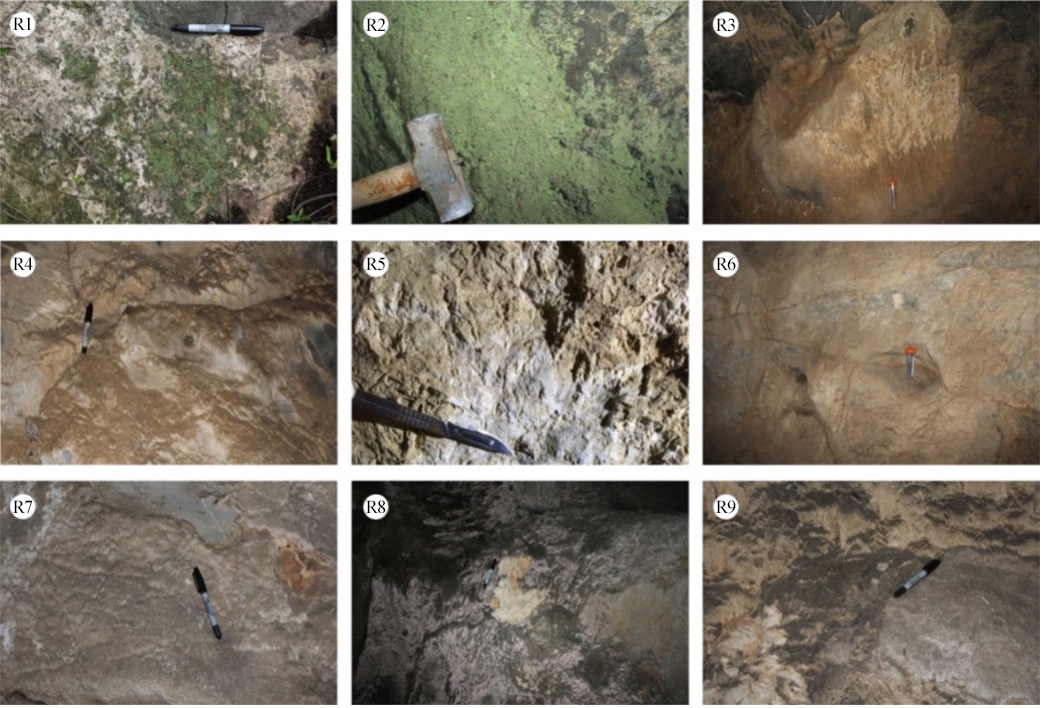


Supplementary Figure 2. Images of sampling sites of weathered rocks in the Heshang Cave, P R China. The nine sampling sites had a high degree of weathering and obvious stratification characteristics, and the outermost part of the rock wall was a loose thin layer; the surface layer underneath was a white crust-like mineral aggregate connected to the bedrock, called the crust layer; and there were also some loose mineral particles at the connection between part of the hard crust layer and the bedrock or in the fissures of rock. R1, R2 and R3 were in the photic zone, R1 and R2 were close to the hole, the rock surface was covered with green lichens with underlying adhered black minerals; R4, R5 and R6 were in the twilight zone, most of them were brownish rock walls; R7, R8 and R9 were in the aphotic zone, and with black biofilm patches at R8 and R9.


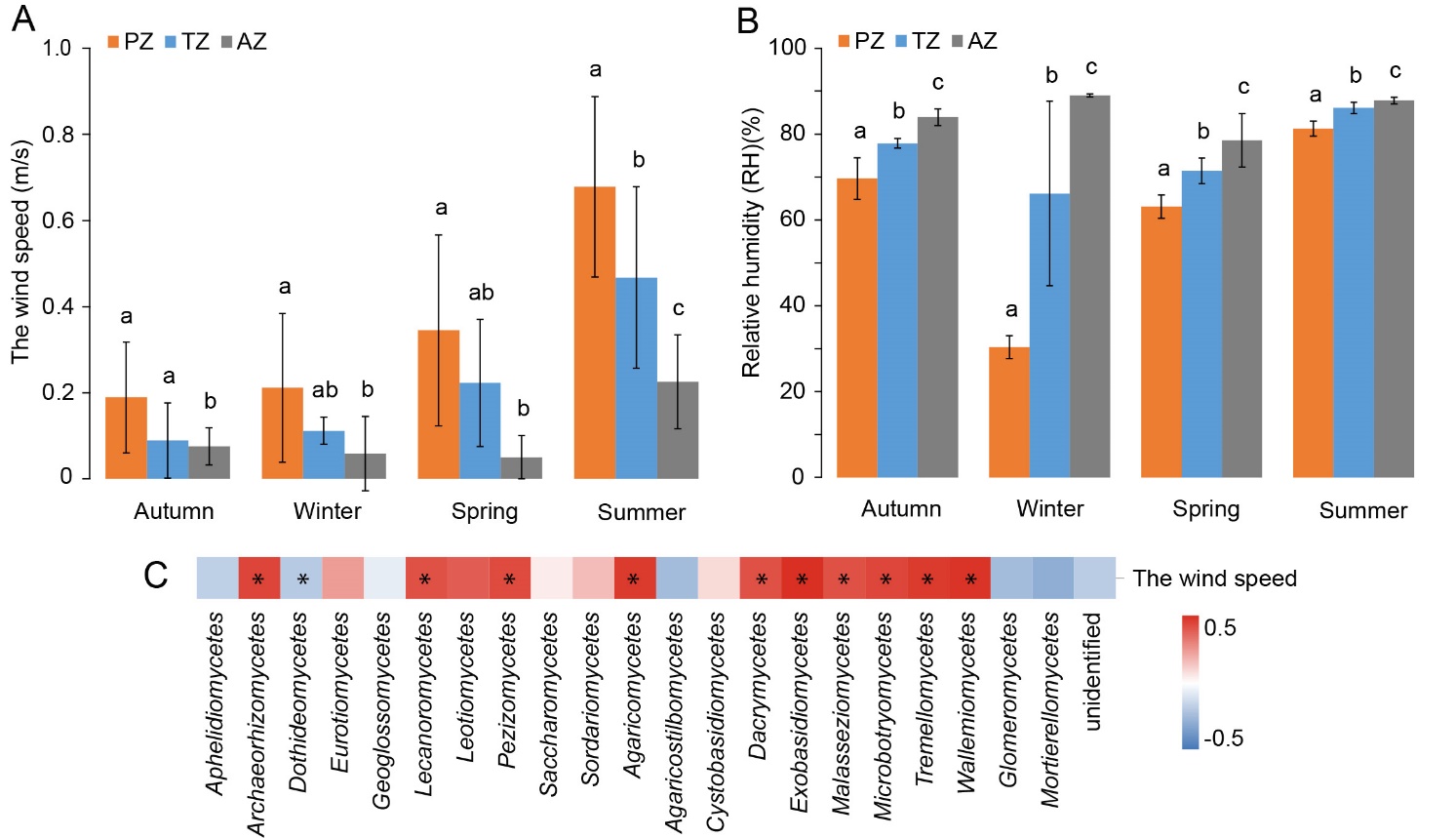


Supplementary Figure 3. The average wind speed (A) and average relative humidity (B) at different light zones in different seasons in the Heshang Cave, P R China. Different letters (a- c) above the bars show significant difference (*P* < 0.05) among groups based on one-way ANOVA. (C) Heatmap of fungal class with the average wind speed and average relative humidity in the Heshang Cave. The mark with * indicates significant correlation (*P* < 0.05).


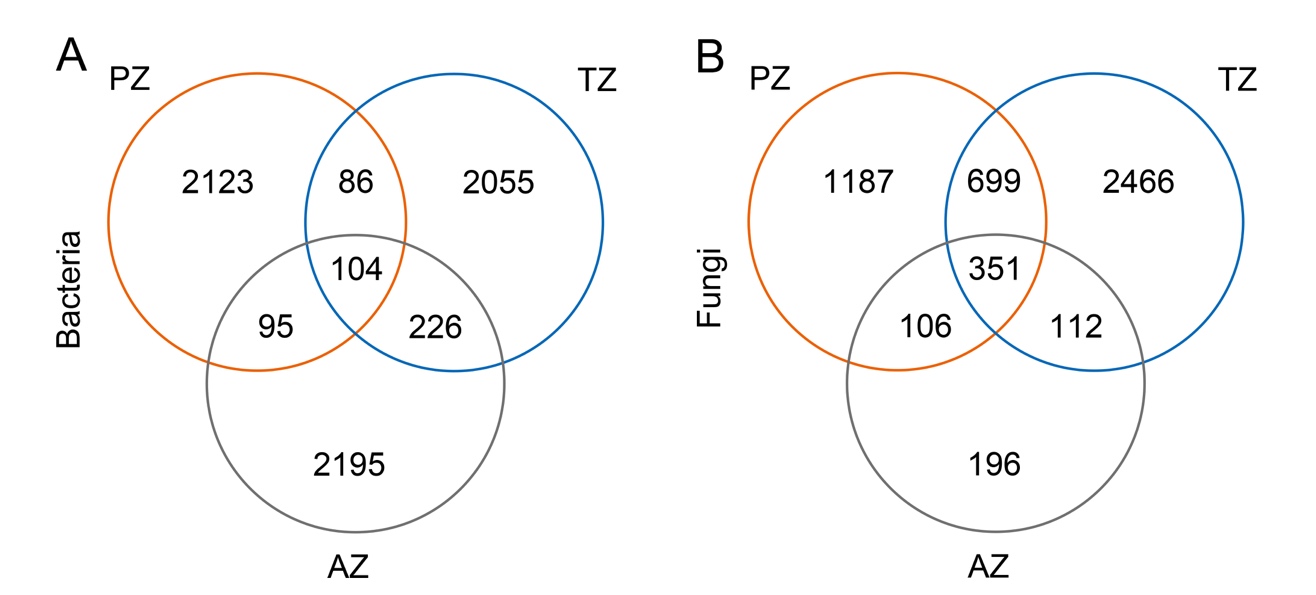


Supplementary Figure 4. Venn diagrams of displaying bacterial ASVs (A) and fungal OTUs (B) in the photic zone (PZ), twilight zone (TZ) and aphotic zone (AZ) in Heshang Cave.


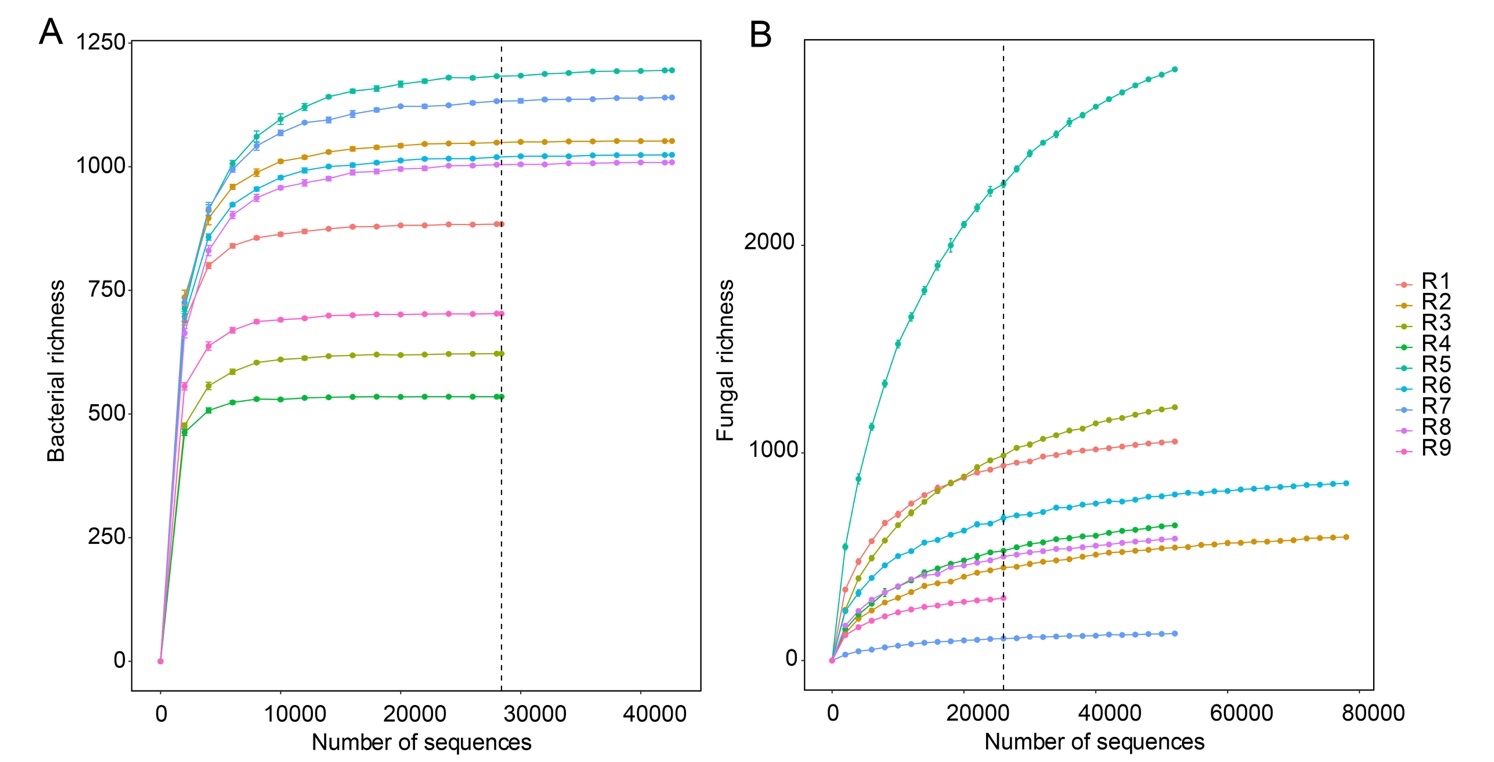


Supplementary Figure 5. Rarefaction curves of bacteria (A) and fungi (B) at different sampling sites in the Heshang Cave, P R China.

Supplementary Table 1. Mineral composition of weathered rock samples along the Heshang Cave (wt%), Hubei province

|  | quartz | dolomite | calcite | amorphous | | mgcalcite | gypsum | illite | hydroxylapatite | fluorapatite | whitlockite |
| --- | --- | --- | --- | --- | --- | --- | --- | --- | --- | --- | --- |
| PZ1 | 35.4 | 12.2 | 9.9 | | 42.5 | ND | ND | ND | ND | ND | ND |
| PZ2 | 31.8 | ND | ND | | 11.2 | 57 | ND | ND | ND | ND | ND |
| PZ3 | 86 | ND | ND | | 14 | ND | ND | ND | ND | ND | ND |
| PZ4 | ND | ND | 50.3 | | 11.1 | ND | ND | ND | ND | ND | ND |
| PZ5 | 73.6 | 7.1 | 8.6 | | 10.7 | ND | ND | ND | ND | ND | ND |
| PZ6 | 53.9 | 4.9 | ND | | 15.1 | 26.1 | ND | ND | ND | ND | ND |
| PZ7 | 29.2 | ND | ND | | 7.4 | 63.3 | ND | ND | ND | ND | ND |
| TZ1 | 68.9 | 6.1 | 5 | | 12.9 | ND | ND | 7.1 | ND | ND | ND |
| TZ2 | 27.4 | 5.2 | ND | | 22.8 | ND | ND | ND | 22.4 | 22.4 | ND |
| TZ3 | 33.9 | 59.4 | ND | | 6.6 | ND | ND | ND | ND | ND | ND |
| TZ4 | 35.8 | 9.4 | ND | | 21.2 | ND | ND | ND | 33.6 | ND | ND |
| TZ5 | 21 | 30.4 | 20.4 | | 12.2 | ND | ND | ND | 16.1 | ND | ND |
| TZ6 | 60.4 | 15.4 | ND | | 15.4 | ND | ND | 8.9 | ND | ND | ND |
| TZ7 | 21.6 | 11.1 | ND | | 24.1 | ND | 3.6 | ND | 19.8 | 19.8 | ND |
| TZ8 | 50.5 | 33 | 5.6 | | 10.8 | ND | ND | ND | ND | ND | ND |
| AZ1 | 23.5 | 3.1 | ND | | 23.4 | ND | ND | ND | 29.6 | ND | 20.3 |
| AZ2 | ND | ND | ND | | 22.4 | ND | ND | ND | 63 | ND | 14.6 |
| AZ3 | 16.5 | 65.1 | 13.9 | | 4.5 | ND | ND | ND | 0 | ND | ND |
| AZ4 | 43.2 | ND | ND | | 10.1 | ND | 24.8 | ND | 22 | ND | ND |
| AZ5 | ND | ND | ND | | 21.5 | ND | ND | ND | 42.7 | 35.9 | ND |
| AZ6 | 39.5 | 16 | ND | | 22 | ND | 2.6 | ND | 19.9 | ND | ND |
| AZ7 | 23.8 | 70.6 | ND | | 5.6 | ND | ND | ND | ND | ND | ND |
| AZ8 | 43.2 | ND | ND | | 21.7 | ND | ND | ND | ND | 35.1 | ND |

PZ1-PZ8: photic zone; TZ1-TZ8: twilight zone; AZ1-AZ8: aphotic zone; ND: Not detected

Supplementary Table 2. The wind speed (m/s) at different sampling sites in different seasons

in the Heshang Cave

| Rock sites | Oct.2020 | Jan.2021 | Apr.2021 | Jul.2021 |
| --- | --- | --- | --- | --- |
| R1 | 0.3 ± 0.1 | 0.3 ±0.3 | 0.6 ±0.1 | 0.7 ±0.1 |
| R2 | 0.1 | 0.2 ±0.1 | 0.3 | 0.8 ±0.1 |
| R3 | 0.2 | 0.1 ±0.1 | 0.1 | 0.5 ±0.1 |
| R4 | 0.2 | 0.1 | 0.4 ± 0.1 | 0.7 ±0.2 |
| R5 | 0.0 ± 0.1 | 0.1 ± 0.1 | 0.3 ± 0.1 | 0.3 ±0.1 |
| R6 | 0.0 ± 0.1 | 0.1 | 0.0 ± 0.1 | 0.3 ±0.1 |
| R7 | 0.1 ± 0.1 | 0.0 | 0.1 ± 0.1 | 0.4 ±0.1 |
| R8 | 0.1 ± 0.1 | 0.0 ± 0.1 | 0.0 ± 0.1 | 0.1 ±0.1 |
| R9 | 0.1 ± 0.1 | 0.1 ± 0.2 | 0.1 ± 0.1 | 0.2 ± 0.1 |

Supplementary Table 3. The relative humidity (%) at different sampling sites in different seasons

in the Heshang Cave

| Rock sites | Oct.2020 | Jan.2021 | Apr.2021 | Jul.2021 |
| --- | --- | --- | --- | --- |
| R1 | 65.3 ± 1.3 | 28.9 ± 3.2 | 60.8 ± 1.4 | 82.1 ± 0.1 |
| R2 | 68.0 ± 3.9 | 29.5 ± 1.7 | 62.1 ± 2.3 | 80.3 ± 2.7 |
| R3 | 75.7 ± 1.4 | 32.7 ± 3.1 | 66.4 ± 1.3 | 81.5 ± 2.3 |
| R4 | 78.5 ± 1.8 | 36.6 ± 3.3 | 68.8 ± 1.5 | 84.8 ± 0.9 |
| R5 | 76.9 ± 1.0 | 75.2 ± 1.3 | 70.2 ± 2.1 | 85.9 ± 0.5 |
| R6 | 78.1 ± 0.7 | 86.7 ± 1.0 | 75.2 ± 1.0 | 87.7 ± 0.2 |
| R7 | 80.8 ± 0.8 | 88.7 ± 0.7 | 68.8 ± 2.8 | 88.3 ± 0.3 |
| R8 | 84.1 ± 0.2 | 89.2 ± 0.1 | 77.7 ± 0.6 | 88.5 ± 0.2 |
| R9 | 85.4 ± 0.4 | 89.0 ± 0.3 | 83.8 ± 0.8 | 87.3 ± 1.1 |

Supplementary Table 4. Alpha diversity index of bacteria and fungi in different sampling zones along the Heshang cave

| Locations | Light zone | Bacterial Shannon | Bacterial ACE | Fungal Shannon | Fungal ACE |
| --- | --- | --- | --- | --- | --- |
| R1 | PZ1 | 8.28 | 7.48 | 6.08 | 13.09 |
| R1 | PZ2 | 8.69 | 8.70 | 6.28 | 11.90 |
| R2 | PZ3 | 8.37 | 7.60 | 3.58 | 8.35 |
| R2 | PZ4 | 7.56 | 4.18 | 4.57 | 8.05 |
| R2 | PZ5 | 8.55 | 8.60 | 4.73 | 8.30 |
| R3 | PZ6 | 8.05 | 7.72 | 4.82 | 14.13 |
| R3 | PZ7 | 7.76 | 6.15 | 4.49 | 12.08 |
| R4 | TZ1 | 8.02 | 4.98 | 4.61 | 11.97 |
| R4 | TZ2 | 7.58 | 4.76 | 3.04 | 8.43 |
| R5 | TZ3 | 8.07 | 8.40 |  |  |
| R5 | TZ4 | 7.84 | 8.87 | 8.62 | 24.09 |
| R5 | TZ5 | 8.42 | 10.60 | 5.74 | 9.73 |
| R6 | TZ6 | 8.02 | 6.00 | 4.95 | 9.36 |
| R6 | TZ7 | 7.59 | 8.03 | 3.90 | 11.91 |
| R6 | TZ8 | 7.86 | 6.60 | 4.76 | 8.12 |
| R7 | AZ1 | 7.68 | 5.88 | 1.24 | 5.78 |
| R7 | AZ2 | 8.37 | 7.94 | 1.15 | 3.81 |
| R7 | AZ3 | 8.81 | 10.50 |  |  |
| R8 | AZ4 | 7.67 | 6.48 | 4.46 | 11.01 |
| R8 | AZ5 | 7.70 | 5.36 |  |  |
| R8 | AZ6 | 8.56 | 9.79 | 4.31 | 6.84 |
| R9 | AZ7 | 7.64 | 5.03 | 4.00 | 8.96 |
| R9 | AZ8 | 8.35 | 8.03 |  |  |
